# Supplementary material for: HIVprotI: an integrated web based platform for prediction and design of HIV proteins inhibitors
Source: J Cheminform. 2018 Mar 9;10:12. doi: 10.1186/s13321-018-0266-y (PMC5845081; doi:10.1186/s13321-018-0266-y)
Supplement: Supplementary file 2 — Additional file 2. Source code of HIVProtI web server. [file 13321_2018_266_MOESM2_ESM.zip › HIVprotI_Source-code/hivprotI/weka-3-6-6/doc/weka/core/pmml/FieldMetaInfo.html]

FieldMetaInfo


---


|  |  |  |
| --- | --- | --- |
| **Overview** \| **Package** \| **Class** \| **Tree** \| **Deprecated** \| **Index** \| **Help** \| **Weka's home** | |  |
| **PREV CLASS**   **NEXT CLASS** | **FRAMES**    **NO FRAMES**     **All Classes** |  |
| SUMMARY: NESTED \| FIELD \| CONSTR \| METHOD | DETAIL: FIELD \| CONSTR \| METHOD |  |


---


## weka.core.pmml Class FieldMetaInfo

```
java.lang.Object
  weka.core.pmml.FieldMetaInfo
```

**All Implemented Interfaces:**: java.io.Serializable

**Direct Known Subclasses:**: DerivedFieldMetaInfo, MiningFieldMetaInfo, TargetMetaInfo

---

``` public abstract class FieldMetaInfo extends java.lang.Object implements java.io.Serializable ```

Abstract superclass for various types of field meta
data.

**Version:**
:   $Revision 1.0 $

**Author:**
:   Mark Hall (mhall{[at]}pentaho{[dot]}com

**See Also:**: Serialized Form

---

| **Nested Class Summary** | |
| --- | --- |
| `static class` | `FieldMetaInfo.Interval`             Inner class for an Interval. |
| `static class` | `FieldMetaInfo.Optype`             Enumerated type for the Optype |
| `static class` | `FieldMetaInfo.Value`             Inner class for Values |


**Constructor Summary**

`FieldMetaInfo(org.w3c.dom.Element field)`
  
          Construct a new FieldMetaInfo.


| **Method Summary** | |
| --- | --- |
| `abstract  Attribute` | `getFieldAsAttribute()`             Return this field as an Attribute. |
| `java.lang.String` | `getFieldName()`             Get the name of this field. |
| `FieldMetaInfo.Optype` | `getOptype()`             Get the optype. |

**Methods inherited from class java.lang.Object**

`equals, getClass, hashCode, notify, notifyAll, toString, wait, wait, wait`

**Constructor Detail**

### FieldMetaInfo

```
public FieldMetaInfo(org.w3c.dom.Element field)
```

:   Construct a new FieldMetaInfo.

    **Parameters:**: `field` - the Element containing the field


**Method Detail**

### getOptype

```
public FieldMetaInfo.Optype getOptype()
```

:   Get the optype.

    :   **Returns:**: the optype

---


### getFieldName

```
public java.lang.String getFieldName()
```

:   Get the name of this field.

    :   **Returns:**: the name of this field

---


### getFieldAsAttribute

```
public abstract Attribute getFieldAsAttribute()
```

:   Return this field as an Attribute.

    :   **Returns:**: an Attribute for this field.


---


|  |  |  |
| --- | --- | --- |
| **Overview** \| **Package** \| **Class** \| **Tree** \| **Deprecated** \| **Index** \| **Help** \| **Weka's home** | |  |
| **PREV CLASS**   **NEXT CLASS** | **FRAMES**    **NO FRAMES**     **All Classes** |  |
| SUMMARY: NESTED \| FIELD \| CONSTR \| METHOD | DETAIL: FIELD \| CONSTR \| METHOD |  |


---
